# An Iterative Unsupervised Method for Gene Expression Differentiation

**DOI:** 10.3390/genes14020412

**Published:** 2023-02-04

**Authors:** Olga Georgieva

**Affiliations:** Faculty of Mathematics and Informatics, Sofia University “St. Kliment Ohridski”, 125 Tsarigradsko Shosse Blvd., bl. 2, 1113 Sofia, Bulgaria; o.georgieva@fmi.uni-sofia.bg

**Keywords:** gene expression data, differentially expressed genes, clustering analysis, density-based clustering

## Abstract

For several decades, intensive research for understanding gene activity and its role in organism’s lives is the research focus of scientists in different areas. A part of these investigations is the analysis of gene expression data for selecting differentially expressed genes. Methods that identify the interested genes have been proposed on statistical data analysis. The problem is that there is no good agreement among them, as different results are produced by distinct methods. By taking the advantage of the unsupervised data analysis, an iterative clustering procedure that finds differentially expressed genes shows promising results. In the present paper, a comparative study of the clustering methods applied for gene expression analysis is presented to explicate the choice of the clustering algorithm implemented in the method. An investigation of different distance measures is provided to reveal those that increase the efficiency of the method in finding the real data structure. Further, the method is improved by incorporating an additional aggregation measure based on the standard deviation of the expression levels. Its usage increases the gene distinction as a new amount of differentially expressed genes is found. The method is summarized in a detailed procedure. The significance of the method is proved by an analysis of two mice strain data sets. The differentially expressed genes defined by the proposed method are compared with those selected by the well-known statistical methods applied to the same data set.

## 1. Introduction

For several decades, intensive investigations for revealing and understanding genes’ role in organisms’ lives is a research field of scientists in different specialties. A part of these efforts is the analysis of gene expression data for searching relations in the genes’ activities. The expression data are numerical tables obtained through well-established technologies that have been significantly improved in the last years. The older technology for microarray data outcome does not reach the high accuracy of the contemporary technologies named next generation sequencing. The new technology provides larger volumes of DNA-Seq and RNA-Seq data. Thus, next generation sequencing technologies significantly increase the opportunity for effective research and gathering knowledge for existing dependencies of the genes’ activity.

Regardless of the technology used, it results in a gene expression table. Each row of the table corresponds to a particular gene and each column to a sample. The genes in the table consist of a part or whole genome of a given organism. The sample data concerns the specific research aim. If they comprise samples of distinct strains, genes that are differently expressed are of interest. In the case of samples that correspond to certain environmental conditions, the genes responsible for the conditions’ activities have to be identified. The challenge is this huge amount of data, which in case next generation sequencing technologies contain up to several hundred thousand numbers that must be processed by effective methods of data analysis. The aim is to reveal information about unknown biological dependences. The information gained helps in answering the main questions of life, such as how genome sequence specifies the forms and functions of the organisms for the remarkable diversity of life or helps in understanding the evolutionary mechanisms of the organisms [1,2,3,4]. In this direction, the approach of finding groups of genes that act equally and identifying genes unknown in their biological response or finding genes involved in certain processes is promising. For instance, a specific type of cancer with respect to a group of genes responsible for it is valuable to research. Classification obtained from gene expression analysis can be used for more precise tumor diagnosis and its effective treatment [1].

The marked problems need to discover groups of genes that behave similarly. The task could be solved in a supervised manner if preliminary knowledge about the interested groups exists. However, in most cases, it is searched for unknown data partitioning that needs the application of sophisticated statistical or machine learning methods.

A survey of statistical methods applied to identify a set of transcripts that are differentially expressed between distinct experimental conditions with the goal of providing a comprehensive guide when choosing appropriate metrics for RNA-Seq statistical analyses is provided in [5]. Gaussian mixture modeling was investigated to detect and characterize bimodal gene expression patterns across cancer samples to explore the hypothesis that cancer mutations are likely to cluster with specific dichotomous shifts in the expression of the genes [6]. Despite the statistical methods being widely applicable, they suffer in difficulty ensuring the statistical distinguishableness of the result.

The difficulty to find well-separated groups of genes is an incentive to explore other approaches in addition to statistical ones. The application of machine learning methods was investigated and improvement in patient outcomes is shown in [7,8]. The obtained results indicate that these algorithms can effectively differentiate healthy subjects and affected patients. Successful implementation of tasks of expression differentiation is demonstrated in [9]. Additionally, it is shown that convolutional neural networks achieves the best results among eight explored deep learning methods for cancer classification [10]. Various machine learning models of voting classifiers are built and compared in terms of their ability to human protein prediction [11]. The ability of clustering analysis to deal with various tasks of genes’ activity understanding has to be underlined to identify biomarkers and yield computational predictive models [12] or a set of reproduction operators to facilitate the exchange of grouping information between chromosomes [13].

A specific task of gene expression analysis is the aim to detect genes that are distinguishable by their expression. These are genes that act differently in the same strains. By solving the problem, the genes responsible for certain disease states can be discovered or it is possible to find differences between two species of strains. In searching for an appropriate task solution, several methods for RNA-Seq data analysis according to their expression levels based on statistical data analysis have been explored [14,15,16]. The differentially expressed genes have been selected according to a value of a predefined threshold. Their systematic and deep comparison performance shows that there is no good agreement among the applied methods as, aside from the commonly identified genes, each statistical method detects additional genes not identified by the others [15]. Thus, only 570 genes were recognized as significant by four different methods, namely DESeq, DESeq2, edgeR, and the limma method, which is closely related to the to the t-test. Each of these methods has been chosen as a representative of a subgroup of the group of fifteen statistical methods. By taking the advantage of the unsupervised data analysis, an iterative clustering procedure that finds the differentially expressed genes was recently introduced [17], showing that results are comparable with those of the statistical methods. The difficulty of the selection problem due to a large number of indistinguishable genes is solved by an iterative procedure with parameters that are a subject of a preliminary choice. However, the research lacks a well-ground choice in both the implemented clustering algorithm as well as the procedure parameters. Another drawback is that differentially expressed genes are found only by the difference in their average expression value not accounting for the variation level.

The present paper aims to improve our newly proposed method [17] for gene expression differentiation in several directions. First, a comparative study of the clustering methods applied for gene expression analysis explicates the choice of the clustering algorithm implemented in the introduced method. Second, an investigation of different distance measures is provided to reveal those that increase the efficiency of the method in finding the real data structure. Third, the method is improved by incorporating an additional aggregation measure based on the standard deviation of the expression levels. Its application increases the gene distinction and a new amount of differentially expressed genes is found. Fourthly, the method is summarized in a detailed step procedure. The significance of the method is proved by the analysis of two mice strain data sets. The differentially expressed genes defined by the proposed method are compared with those selected by the well-known statistical methods applied to the same data set.

## 2. Clustering for Gene Expression Data Analysis

Machine learning methods based on an unsupervised approach, such as cluster analysis, present appropriate features for gene expression differentiation and grouping [18,19]. Clustering analysis divides a collection of data into groups and does not need a reference model. The data are similar to each other within the cluster and different from the data of other clusters. There are a large number of clustering algorithms that differ in how they solve the two main issues of the task [12,18,20,21]. First, what is the proper similarity measure to assess the data proximity, and second, what procedure to use in order to find the data groups. The first one is to define the shape of the determined clusters. The clustering procedure is governed by the incorporated partition criterion that in fact imposes the data structure. However, there is no prescription for which criterion and how to choose [18,19]. Despite the large diversity of clustering methods, they could be grouped according to the implemented clustering technique. Three main groups could be underlined according to their abilities to deal with gene expression data analysis.

### 2.1. Hierarchical Clustering for Gene Expression Analysis

The most applicable clustering method for gene expression analysis is hierarchical clustering [2,19]. Each cluster is built by smaller clusters, forming a tree-shaped data structure. Agglomerative hierarchical clustering starts with single-gene clusters and successively joins the closest clusters until all genes have been joined into the supercluster. The opposite strategy starts with all data collected in a single cluster and further divides them into smaller groups. The agglomerative hierarchical algorithm is the most widely used for gene expression analysis [2]. The important question is the cutting level of the dendrogram in order to obtain the right clusters. Additionally, a family of clustering methods is known according to the implemented linkage function. Due to the existing indistinguishability, different linkage functions have to be explored to establish good clustering.

The establishment of the hierarchical clustering method as a widely used method for clustering gene expression data is owed to its visibility features. The clustering is presented in a dendrogram. They are also given in a colored view way that became a standard for visualization of the gene expression data.

### 2.2. Objective Function Clustering for Gene Expression Analysis

Objective function clustering applies a criteria function that measures the quality of the partitioning. The optimal value of the criteria function determines the data grouping. The most used objective function clustering algorithm is *k*-means which divides the data into a predetermined number of *k* clusters. The algorithm identifies the clusters according to their representatives—the cluster centers. Data points are assigned to a cluster on the basis of the distances from the centroids. An application for gene expression analysis is demonstrated in [9,19].

A family of objective function algorithms based on *k*-means has been proposed. The major question of their application is how many clusters actually exist and thus how to initialize the algorithm. The question is the subject of additional research. One possible approach is to initialize with *k* randomly chosen cluster centroids, and each gene is assigned to the cluster with the closest centroid. Another good strategy is seeding prototype centroids with the eigenvectors identified by principal component analysis performed optimally for genes of yeast bacteria [7]. Subtractive clustering was successfully implemented to determine the number of clusters of gene expression soil bacterium data [22].

The fuzzy variant of the *k*-means algorithm, fuzzy C-means, shows a large advantage for gene expression analysis, as it finds clusters that are overlapped. Thus, it reflects the real relationship between genes pointing to distinct regulations and features of each gene’s function [7,22].

The self-organizing map (SOM) methods are underlined to join this group of clustering methods. SOM methods find clusters, which are organized into a grid structure. The search procedure follows the same idea of proximity assessment of the input vector [1,20].

### 2.3. Density-Based Algorithms for Gene Expression Analysis

The density-based clustering searches for dense areas in the data space. It is not necessarily to generate clusters explicitly, but instead to show the bunches of data that form cluster structure. These algorithms are a good way to separate clusters from the noise. They allow for a centralized description of irregularly shaped clusters in a data set with high dimensions to identify outliers as data points with low cardinality [8,23]. By such an algorithm implemented in gene-based clustering, the dense and not dense areas of data are revealed to explain complexes and patterns of the gene associations [23]. A density-based clustering algorithm named DBSCAN has a simple scheme for cluster detection using a matrix of pairwise distances to find outliers and core points. Its complex variation shows better efficacy [24] as an alternative density-based clustering algorithm implementation.

It should be underlined that other methods different from the ones discussed above have been proposed in recent years for gene expression analysis. Pattern-based clustering algorithms form clusters by objects, whose attributes present a difference of changes of the values of the attributes smaller than a threshold value. Another workable idea was to construct and apply a clustering algorithm in an iterative manner, which is a helpful strategy in case of a vast amount of data. It is practicable to process data by subdividing the genes into a smaller number of categories and then analyzing the obtained groups [20].

## 3. Method of Gene Expression Differentiation Based on Iterative Clustering Analysis

**Figure 1:**
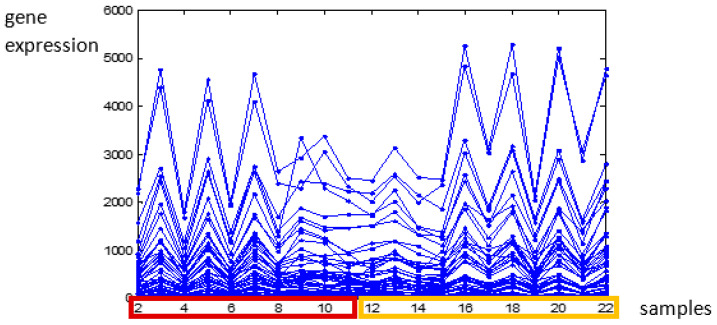
Profiles of the first 100 most differentially expressed genes of the two mice data sets. The first 10 samples (in red) are one mouse strain and the rest 11 (in yellow) are another strain.

The gene expression differentiation problem is different from the general task of gene expression analysis as instead of finding gene groups, it tries to find genes that have significantly different activity in their expression levels. The difficulties in solving this task are as the following:

- Assessments based on statistical analysis are rather hard. The number of genes is large and they lack significant apartness according to the estimated *p*-values. Thus, no precise conclusion about the genes’ distinguishing could be performed.
- Another particularity of the data is that often the number of interested genes is quite smaller than the whole data amount. In addition, the searched genes appeared mostly as outliers than as a representative and compact group that could be interpreted.
- The difficulty of the gene separation is a result of the existing difference in the gene expression of the samples of a given strain that in some cases is larger than the expression between the compared strains. This fact is illustrated in Figure 1, where profiles of gene expressions of two mice strains are given. The data are provided in [25] and will be described in more detail in fourth section. It is clear to see that the deviation in the gene expression within the same strain samples is larger than between the expressions of the two strains.

According to these observations, it could be concluded that an alternative method rather than a statistical one is valuable to be investigated. Unsupervised methods, such as clustering analysis, are promising due to the ability to deal with no reference model and with unbalanced groups of data. Another important conclusion concerns the impossibility to distinguish genes within the samples of a strain. It imposes a solution based on some aggregated measure to represent the expression levels of a gene.

Following the above conclusions about the gene expression data and knowing the abilities of the distinct types of clustering methods, the method of iterative gene expression differentiation is substantially improved to affirm it as an appropriate method for gene differentiation.

### 3.1. Iterative Clustering Analysis for Gene Expression Differentiation

The method of gene expression differentiation uses the average value of the gene expression of the samples of a particular strain as an aggregated measure of the genes’ activity. Comparing the average values of each gene for two investigated strains, we could expect that for the genes that behave equally the respective average values remain close whereas, for the differently expressed genes, the mean values differ. The latter are differentially expressed genes that we are interested in. However, their direct comparison will not give a reliable result, as we do not have a threshold value to separate the two groups. It is necessary to apply an additional assessment that could distinguish the not separable data from those that are much different for the two strains. A valuable distinguishing could be performed with a proper clustering algorithm.

In the present work, we introduce the standard deviation of the expression values of a gene as a second aggregating measure that could give additional information in terms of genes’ distinguishing. The rationale for this is the fact that even if the two compared average values are similar, the genes could behave differently if their deviation from the mean value differs at large.

In accordance with this idea, we are looking for a clustering algorithm to enable us to distinguish the two kinds of data—similarities and outliers. A good choice is the widely applicable DBSCAN algorithm [24], which groups data based on their density and finds clusters, as well as outliers. It computes clusters iteratively by exploring the closeness of each data to the others in the accepted radius. The algorithm is basic and scalable to large data sets. Variations of the algorithm discover clusters with different densities and kernel functions by exploring additional information for the algorithm parameters [26,27]. The reasonability to use its sophisticated version in our case is questionable as we are interested mostly in the outliers. Additionally, the core clusters are expected to be commensurate with as far as all they are along the equivalent area.

The algorithm is applied to the data formed by the average expression values of each gene for the different strains. Using this algorithm, we can separate genes densely scabbed around the equivalence line of the data space from the outliers that are away from this area. The outliers are the data of the genes we are searching for.

DBSCAN clustering uses a matrix of pairwise distances between data. It finds the number of outliers and core points. The clustering is accomplished based on a threshold radius *r* for neighborhood search and a minimum number of neighbors *Nmin* required to identify a core point. The two parameters are subject to off-line investigation and fully depend on the structure of the data space. The default measure for data range estimation is Euclidian distance.

**Figure 2:**
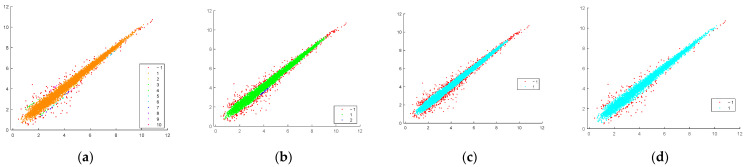
Results of DBSCAN clustering applied to the average values of the genes of the two mice strains (**a**) *r* = 0.2 and *Nmin* = 5 discovers 153 differently expressed genes; (**b**) *r* = 0.2 and *Nmin* = 15 discovers 420 genes; (**c**) *r* = 0.2 and *Nmin* = 20 discovers 593 genes; (**d**) *r* = 0.4 and *Nmin* = 20 discovers 154 genes. Discovered core clusters are enumerated in the legends and outliers (red dots) are marked by “−1”.

Clustering by the DBSCAN algorithm is applied to Bottomly’s reduced data set [25] (Figure 2). Each graphic of the figure presents the scatter plot of the averaged values of the gene expressions, where the first dimension corresponds to one mouse strain and the second dimension to the other strain. By varying the clustering parameters *r* and *Nmin,* different numbers of core clusters (compact clusters in the equivalence area) and outliers (red dots) are discovered for each data set. The outliers that surround the compact data group(s) are the data of differently expressed genes. For small cluster radius *r* and low *Nmin,* the number of discovered core clusters is relatively high and the number of outliers is small (Figure 2a). By increasing *Nmin,* more outliers are identified (Figure 2b,c). The radius *r* is a sensitive parameter. When it doubles, the number of differently expressed genes decreases drastically (Figure 2c,d).

A problem with clustering the entire data set is accounted for due to the enormous number of similarly behaving genes. The algorithm reveals a limited amount of differently expressed genes (Figure 2). Compared to those obtained by the statistical analysis results [15], they are less. In order to improve the distinctiveness of the interested genes and thus their disclosure, the clustering can be applied not to the whole set at once, but by sequentially separating subsets of the data. These data subsets must have the same volume and here we call them data batches. Based on this idea, an iterative clustering scheme is proposed that significantly increases the number of discovered genes.

The whole amount of data is divided into batches that each comprise an equal number of gene expression data. The clustering procedure incorporates the DBSCAN algorithm applied iteratively to the data of each batch. The outliers discovered in each batch are added to form a common set of differently expressed genes.

This scheme needs to solve two important questions in advance, such as data preprocessing and setting the values of the parameters needed for the iterative clustering procedure.

### 3.2. Data Preprocessing

Two main obstacles have to be tackled before clustering. First, logarithm transformation is needed in order to solve the scalability problem, as some of the gene expression values are very large with respect to others. The other problem is that there are zero expression values seen for samples of some genes as they were not active or their expression has been not properly measured. In order to ensure the logarithm calculation, filtering for removing genes with zero activity value is a requisite.

### 3.3. Method Parameters

The choice of the cluster distance is important as it governs the found cluster shape. Other key clustering parameters are the threshold radius *r* for a neighborhood search and a minimum number of neighbors *Nmin* required to identify the core points. The iterative implementation of DBSCAN requires setting the number of data that form a batch. In the present study, by exploring different parameter values, we gain more information according to the method’s applicability.

Due to the similarity in the genes’ behavior as in the case of whole data set processing, it could be expected that the data of each batch form a large compact group along the equivalence area. This area is rather oblong compared to a spherical one. It suggests that clusters are not Euclidean. This observation requires further research for selecting an appropriate proximity measure. Our investigation shows that by applying the Mahalanobis distance, more differentially expressed genes could be discovered in comparison to the Euclidean distance search. In this paper, we extend this investigation by exploring other distance measures and assessing them in terms of their genes’ separation abilities (Section 4).

The threshold radius *r* and the minimum number *Nmin* are difficult to set in advance, as they depend on the density of the data. The threshold radius *r* should not be less than the minimum distance of data pairs. Here, the values of *r* and *Nmin* are determined in searching to increase the number of the discovered outliers.

The number of genes that form a batch is another problem to be solved. A rather large batch could make it impossible to detect all outliers, as in the case of whole data set clustering. Conversely, too small of a batch could embarrass the detection of core clusters and thus the right distinguish between equivalently and differentially expressed genes. Our experience signifies that several hundred data in a batch could produce acceptable results. The application results obtained here show that it is worth exploring different batch volumes in order to find a reliable quantity of differently expressed genes.

Once the method parameters were determined, they are applied to each data batch according to the accepted iterative clustering scheme.

### 3.4. Procedure of Iterative Clustering Method for Gene Expression Differentiation

**Figure 3:**
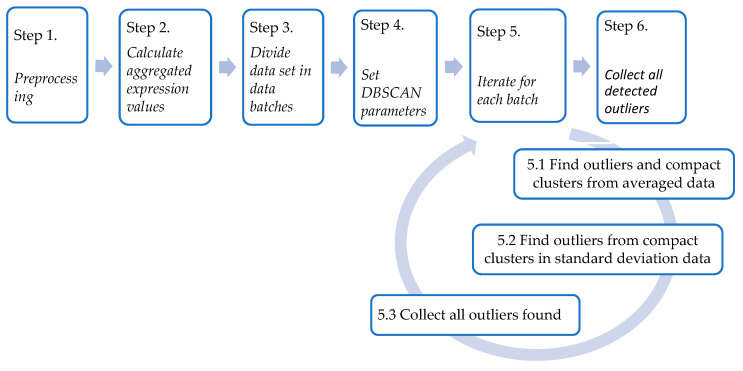
The procedure of the iterative clustering method for gene expression differentiation.

The method procedure is summarized, in detail, in the following steps and illustrated in Figure 3:

Step 1: *Preprocessing*

Filtering for removing genes with zero activity values and log transforming the expression values.

Step 2: *Calculate the aggregated expression values*

Calculation of average values and standard deviation values of expression levels of each gene for each of the compared strains.

Step 3: *Divide the data set into data batches*

Determine the data batches to divide the data set into equal data batches.

Step 4: *Set DBSCAN parameters*

Set a clustering measure that searches for oblong clusters. The two parameters of the algorithm have to be fixed to the threshold radius *r* and the minimum number of data that form a cluster *Nmin.* As there is no strong prescription, their values could be defined by accounting for the specificity of the data set.

Step 5: *Iterate for each batch:*

5.1 Apply the DBSCAN algorithm to the space formed by the average values of the genes of the two strains to extract both outliers and compact clusters.

5.2 Apply the DBSCAN algorithm to the space defined by the standard deviation values of the genes that are in the compact clusters and extract the outliers.

5.3 Collect all outliers found in steps 5.1 and 5.2.

Step 6: *Collect all detected outliers*

Add outliers of each batch to determine the set of differentially expressed genes.

## 4. Application Results and Discussion

The proposed method is applied to an open data set. Conclusions are presented based on the results compared with statistical data analysis for gene expression differentiation of the same data.

### 4.1. Data Set

The iterative procedure is applied to the gene expression data set of samples of two mice strains—ten of strain C57BL/6J and eleven of strain DBA/2J. The raw data available from the ReCount online resource [25] were filtered to represent 13932 genes having non-all-zero rows [15]. As there were zero expression values after filtering the preprocessing step of the method, a reduced data set of 9196 genes remains. After log transformation, the data is further processed.

### 4.2. Analysis of Clustering Parameters

By varying the clustering parameters of different splittings into two groups, the group of outliers (differently expressed genes) and genes that are equivalent in their behavior could be found. First, we explore different distance metrics in an attempt to find the best method parameters (Table 1). For this, the accumulated group of differently expressed genes marked by *ML* is compared with gene groups separated by statistical data analysis by four methods—t-test, edgeR, limma, and DESeq2. The number of discovered genes by each statistical method given in [15] is presented in the first (sub)column of the respective method column. The number of the genes discovered by our method that are common for the respective statistical method is given in the second (sub)column. The last column of the table, “*ML* all data”, consists of the total amount of differently expressed genes that are identified by the proposed iterative clustering method.

Several distance metrics have been investigated and those that produce good results are presented and discussed here. Both Euclidean distance, as well as Minkowski distance with a value of its parameter *p* = 2 (or close to 2), give relatively good results according to the separated clusters. However, as they form spherical clusters that do not correspond to the data structure, it is a prerequisite to distort the separation result by mixing some outliers with the selected core clusters. The distances that produce differently shaped clusters are more valuable with respect to the real data structure. These are Mahalonobis distance and Minkowski distance with a smaller value of parameter *p*. The distances between Cityblock and Chebishev are also investigated. By varying DBSCAN parameters different amounts of genes are discovered, and the best results of each distance investigated for each method are presented in Table 1. Despite the fact that Minkowski distance with a small value of *p* = 0.5 presents the best result according to commonly identified genes with the respective statistical methods, the preference is given to Mahalanobis distance clustering. The oblong clusters through Mahalanobis distance determine the smallest number, 1905 selected genes, compared to 3475 found by Minkovski distance.

In searching for the appropriate batch volume, we explore two different volumes for clustering through best found cluster distance (Table 2). First, the whole amount of data was divided into 18 batches of 511 genes each, except the last one consisting of 508 genes. This batch size is set to be comparable to the number of genes found by the statistical analysis. Further, the batch was set to 1022 and 1016, respectively, for the last one. The differentiation results for batches consisting of 511 and 1022 genes with the appropriate setting of the two parameters, *r* and *Nmin*, are comparable. Certain preferences can be given to clustering at the batch of 511 genes because of the smaller number of all separated genes, the “ML all data” of 1905 genes. On the other hand, the batch of 1022 genes ensures a larger number of differentially expressed genes commonly discovered by the respective statistical methods.

Concerning the rest two parameters, *r* and *Nmin*, it should be underlined that their best values in terms of selecting abilities depend on the applied distance metrics. The minimum number of neighbors *Nmin* is sensitive to the batch size as well (Table 2).

In the next method, the standard deviation is applied as a second aggregated measure to discover additional genes that differ in their variation magnitude. The results shown in Table 3 are obtained from the data separated in the core clusters in accordance with the average measure. A different number of genes are selected varying the standard deviation threshold *Tstd* value. A larger *Tstd* value provides fewer differentially expressed genes. The largest *Tstd* value given in the table for each set of the method parameters corresponds to the minimum number of differentially expressed genes found in the batches. For the results of the next experiments given in the table, the value of *Tstd* was decreased by a step of 0.05 to a reasonable minimum. Further decreasing of the deviation threshold is possible. It results in more selected genes but the biological sense of this should be grounded. The results also show that larger batch size decreases the distinguishing abilities. As a smaller number of genes are identified for the same *Tstd* values.

### 4.3. Visualization of the Results

**Figure 4:**
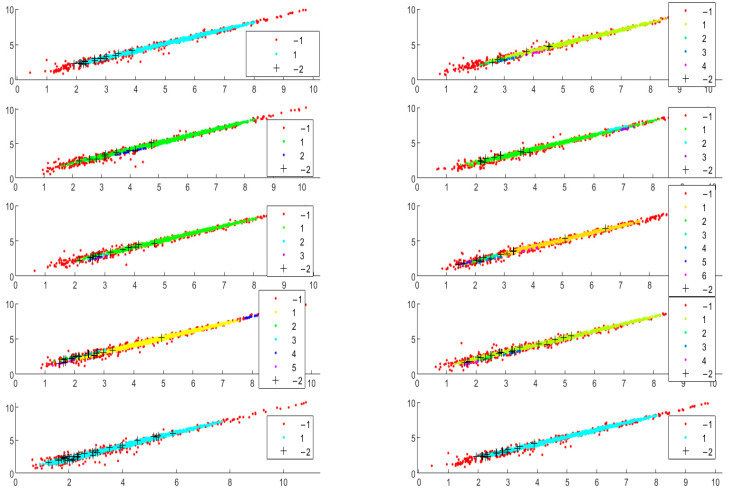
Scatter plots of iterative clustering results via Mahalonobis distance; batch size = 1022, *r* = 0.2, *Nmin* = 10.

The method presents certain visualization abilities. Batch separations are represented as scatter plots, where clusters and outliers are visible. In present, our implementation of the core clusters is colored and enumerated with positive numbers. The outliers found by average expression value are marked by −1, whereas the additionally found data by the standard deviation of the expression values are marked by “+” and enumerated by −2. One particular selection obtained for a set of the method parameters is shown in the scatter plots in Figure 4.

**Figure 5:**
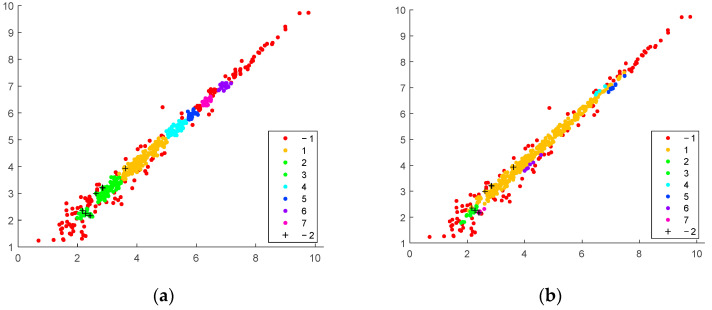
Clustering of the 7th batch and the batch of 511 genes; *Tstd* = 0.3. (**a**) Euclidean distance, 8 *std* genes. (**b**) Mahalonobis distance, 8 *std* genes.

**Figure 6:**
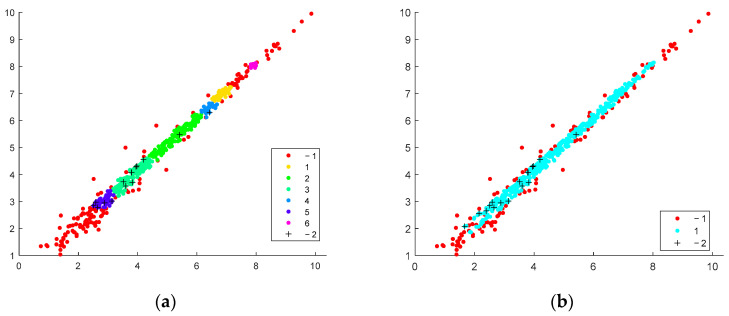
Clustering of the 8th batch, the batch of 511 genes; *Tstd* = 0.3. (**a**) Euclidean distance, 15 *std* genes. (**b**) Mahalonobis, 0.2, 5, 17 *std* genes.

**Figure 7:**
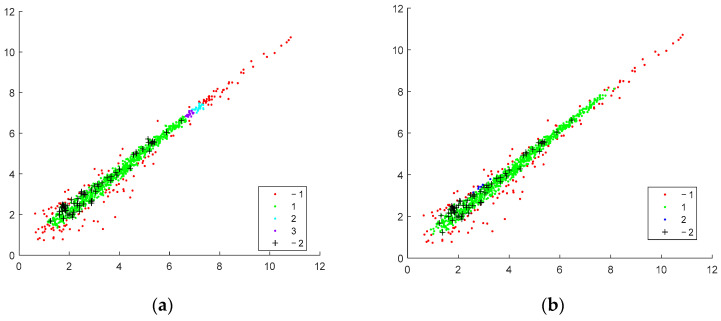
Clustering of the 9th batch, the batch of 1022; *Tstd* = 0.3. (**a**) Euclidean distance, 62 *std* genes. (**b**) Mahalonobis distance, 63 *std* genes.

Detailed visualization of the clustering result with possible interpretation, which could be performed by considering the distinct batch scatter plots. The method’s ability to discover fewer compact clusters along the equivalent area by oblong distance measure is confirmed. In contrast, the spherical distance measure tends to split the data into more clusters. The outliers found using standard deviation measure (*std* found genes) tend to be less for spherical distance than those found by elliptical distance. For instance, the clustering of the same batches with the best-found parameters (Table 1) is presented for Euclidean and Machalonobis distance separation (Figure 5, Figure 6 and Figure 7).

Despite the implemented cluster distance measure in the general case, DBSCAN determines several compact clusters along the equivalent area. Each of these clusters captures genes with very similar behavior that could reveal very specific features. This observation is prerequisite for further knowledge extraction and an opportunity to find genes with different levels of significance revealing new useful information accounting for the biological meaning of the compact clusters found.

The complexity of DBSCAN is measured by consuming *O* (*N log N*) time, with *N* as the size of the data set [26,27]. Relying on this, we can conclude that our method will increase this complexity in a linear way as far as the size *N,* which is presented as m*Nb, where *m* is the number of batches and *Nb* is the size of the batch. However, we should underline that the ability to discover much more outliers is an advantage of the iterative search.

## 5. Conclusions

The paper presents an iterative clustering method for discovering genes that are differentially expressed. The solution is realized by a procedure taking advantage of both density-based clustering and iterative clustering. DBSCAN, as a density-based clustering algorithm is implemented, in which choice is grounded by an analytical review of the ability of the clustering algorithms to deal with gene expression data.

Based on the application of a real data set, the paper makes conclusions about the appropriate method parameters—proper distance measure and batch volume. The best distance measures found are Mahalonobis distance and some parameters of Minkowski distance. Good results are obtained for batches of two different sizes. The results obtained are compared with the results of statistical data analysis applied to the same data set of gene expression of two mice strains.

Additionally, the method incorporates two different aggregated measures of the expression levels—average and standard deviation values used to determine cluster separation of the data space. This gives an opportunity to find genes that are distinguished both by the level of the expression activity and by the level of expression variation if the average values are similar.

The results show that the method is valuable to be applied standalone. However, it could be used in combination with statistical methods for preliminary gene selection within a pipeline of the used algorithms in order to stick their search to a smaller number of genes.

The applicability of the proposed method is a research focus for future work. The procedure should be applied to other data sets to discover differentially expressed genes and to compare results with those obtained by other methods. It is also interesting to research if the method could be used in solving other tasks of gene expression analysis where similarity comparison is needed.

## Figures and Tables

**Table 1:** The number of differentially expressed genes selected through different cluster distances compared with the results of statistical gene differentiation; batch = 511 genes.

Distance	t-test		edgeR		limma		DESeq2		ML All Data
	ttest	ML	edgeR	ML	limma	ML	DESeq2	ML	
Euclidean, *r* = 0.15, *Nmin* = 10	71	71	915	647	736	537	982	648	2848
Mahalanobis, *r* = 0.2, *Nmin* = 5	71	71	915	738	736	611	982	735	1905
Cityblock, *r* = 0.2, *Nmin* = 5	71	70	915	417	736	365	982	407	994
Minkowski, *p* = 2, *r* = 0.2, *Nmin* = 5	71	68	915	286	736	266	982	283	614
Minkowski, *p* = 0.5, *r* = 0.2, *Nmin* = 5	71	71	915	789	736	641	982	807	3475
Chebychev, *r* = 0.1, *Nmin* = 5	71	71	915	667	736	548	982	665	2264

**Table 2:** The number of differentially expressed genes selected via Mahalonobis distance for different batch sizes.

Method Parameters	t-test		edgeR		limma		DESeq2		ML All Data
	ttest	ML	edgeR	ML	limma	ML	DESeq2	ML	
Batch = 511, *r* = 0.2, *Nmin* = 5	71	71	915	738	736	611	982	735	1905
Batch = 511, *r* = 0.3, *Nmin* = 5	71	71	915	527	736	457	982	516	896
Batch = 1022, *r* = 0.2, *Nmin* = 5	71	71	915	584	736	508	982	573	1058
Batch = 1022, *r* = 0.2, *Nmin* = 10	71	71	915	791	736	642	982	787	2050
Batch = 1022, *r* = 0.3, *Nmin* = 10	71	71	915	525	736	455	982	514	891

**Table 3:** The number of selected genes according to standard deviation distinguishing.

Method Parameters	*Tstd*	Number of Selected Genes
Batch size = 511, *r* = 0.2, *Nmin* = 5	0.35	174
	0.3	303
	0.25	560
Batch size = 511, *r* = 0.3, *Nmin* = 5	0.45	84
	0.4	138
	0.35	247
	0.3	417
Batch size =1022, *r* = 0.2, *Nmin* = 10	0.35	147
	0.3	266

## Data Availability

Not applicable.
